# Comparison of native and non-native phone imitation by English and Spanish speakers

**DOI:** 10.3389/fpsyg.2013.00475

**Published:** 2013-07-25

**Authors:** Anne J. Olmstead, Navin Viswanathan, M. Pilar Aivar, Sarath Manuel

**Affiliations:** ^1^Department of Psychology, State University of New YorkNew Paltz, NY, USA; ^2^Haskins LaboratoriesNew Haven, CT, USA; ^3^Department of Experimental Psychology, Universidad Autónoma de MadridMadrid, Spain

**Keywords:** phonetic convergence, speech imitation, non-native speech, Spanish, voice onset time (VOT)

## Abstract

Experiments investigating phonetic convergence in conversation often focus on interlocutors with similar phonetic inventories. Extending these experiments to those with dissimilar inventories requires understanding the capacity of speakers to imitate native and non-native phones. In the present study, we tested native Spanish and native English speakers to determine whether imitation of non-native tokens differs qualitatively from imitation of native tokens. Participants imitated a [ba]–[pa] continuum that varied in VOT from −60 ms (prevoiced, Spanish [b]) to +60 ms (long lag, English [p]) such that the continuum consisted of some tokens that were native to Spanish speakers and some that were native to English speakers. Analysis of the imitations showed two critical results. First, both groups of speakers demonstrated sensitivity to VOT differences in tokens that fell within their native regions of the VOT continuum (prevoiced region for Spanish and long lag region for English). Secondly, neither group of speakers demonstrated such sensitivity to VOT differences among tokens that fell in their non-native regions of the continuum. These results show that, even in an intentional imitation task, speakers cannot accurately imitate non-native tokens, but are clearly flexible in producing native tokens. Implications of these findings are discussed with reference to the constraints on convergence in interlocutors from different linguistic backgrounds.

Interlocutors throughout the course of a conversation tend to adjust their behaviors, becoming more similar with respect to one another. This tendency, sometimes called convergence, has been demonstrated with a number of linguistic (e.g., speech accentedness, Bourhis and Giles, 1977) and non-linguistic behaviors (e.g., foot tapping, Chartrand and Bargh, 1999; postural sway, Shockley et al., 2003). Here, we focus on phonetic convergence, the phenomenon in which interlocutors' productions of sounds and words become more similar to each other throughout a conversation. Specifically, through the use of an intentional imitation task, we examine the constraints that may be placed on phonetic convergence by the phonetic repertoires of individual speakers. Pardo (2006) examined phonetic convergence in pairs of native speakers of American English from slightly different dialect groups. She recorded speakers individually producing a list of target words. She then had pairs of speakers participate in a cooperative map task that elicited spontaneous productions of the same target words. After completing the map task, participants were again recorded on the same set of words. The recorded tokens were presented to a separate group of listeners in an AXB discrimination task. The listeners judged the post-task recordings of a given speaker to be more similar to during-task recordings of their conversational partners than to their own pre-task recordings. This indicates that interlocutors converged during the interaction and that their convergence persisted into the post-task recording session.

While some accounts assume that phonetic convergence occurs automatically because listeners are given to imitating speakers (e.g., Pickering and Garrod, 2004), there have been several findings that demonstrate that convergence is moderated by a number of factors. For instance, Bourhis and Giles (1977) demonstrated that a speaker's expressed attitudes toward an interlocutor had an effect on phonetic convergence. Specifically, they found that while participants speaking Welsh-accented English initially converged to a Received Pronunciation British English speaking experimenter, they diverged from this speaker (they became more Welsh sounding) when the experimenter expressed disdain for the Welsh language. Furthermore, Pardo (2006) found that the extent of phonetic convergence displayed by an individual was different depending on whether the individual acted as an information giver or as an information receiver. In the same study, Pardo also found that the gender of the interlocutors affected convergence patterns (female pairs converged less than male pairs). These studies provide compelling examples of social factors that influence the extent of phonetic convergence, providing evidence that phonetic convergence is not an automatic result of the imitative tendencies of interlocutors.

In this paper, we focus on a different aspect of the conversational setting that has been shown to influence phonetic convergence. Kim et al. (2011) extended the investigation of phonetic convergence by studying interlocutors with different language backgrounds. They tested phonetic convergence in pairs of participants who either shared the same dialect, shared the same language, but spoke different dialects, or were native and non-native speakers of the test language (in this case, native speakers of American English and native speakers of Korean or Chinese, speaking English). They found that phonetic convergence was only likely to occur when participants came from the same language and same dialect groups. Speakers from different native language backgrounds did not converge over the course of the conversation. The authors interpret this result as suggesting that closer linguistic distance between interlocutors facilitates convergence because the existing phonetic repertoires of the interlocutors may be partially aligned to begin with (Babel, 2009).

These findings, taken together, suggest that phonetic convergence within a conversation is not simply a matter of spontaneous perceptual imitation, but is rather complex and dependent upon social and linguistic factors. Indeed, the specific role of imitation in social phonetic convergence is unclear. Pardo et al. (2010), for example, showed that phonetic convergence did not reliably occur in interlocutor pairs wherein one interlocutor was instructed to intentionally imitate the other during a social interaction.

However, despite the unclear role of imitative processes in phonetic convergence, we suggest that the use of phonetic imitation tasks can still inform investigations of phonetic convergence. Principally, we note that in order for convergence to occur, it seems necessary that speakers (1) be flexible in their own productions so as to be able to change them during a conversation and (2) be able to at least approximate the productions of their interlocutor. These requirements suggest that the phonetic repertoires, specifically, the production capabilities of interlocutors play a critical role in phonetic convergence. That is, speakers who are incapable of approximating their interlocutors' speech in an explicit imitation task may fail to show phonetic convergence over short interactions. While similarity in phonetic repertoire may be assumed for interlocutor pairs who come from very similar linguistic backgrounds, the degree of overlap may vary when interlocutors come from vastly different backgrounds. Therefore, one important step in understanding convergence between native and non-native speakers is to determine whether the phonetic repertoire of an individual places constraints on their ability to imitate their interlocutor. Non-social imitation tasks wherein participants are asked to explicitly repeat what is being said provide a useful avenue to study this. Such an approach allows strict control of the imitated tokens as well as a strong manipulation. That is, if participants who are asked to explicitly repeat a given token cannot do so without training, they are unlikely to show convergence in a short-term social task such as a single conversation.

While many imitation studies in both social and non-social situations focus on speakers' vowel productions (e.g., Repp and Williams, 1985; Vallabha and Tuller, 2004; Pardo et al., 2010; Babel, 2011), others have focused on characteristics of consonants. For example, Shockley et al. (2004) had American English speakers shadow words beginning with the voiceless bilabial stop /p/ the voice-onset times (VOT) of which were twice as long as those of naturally produced tokens. They found that imitators' VOTs were significantly longer than baseline when imitating the altered tokens. However, participants' imitations did not reach the extreme VOT values of the presented tokens. Nielsen (2011) expanded on these findings by testing spontaneous imitation of both artificially lengthened VOTs and artificially shortened ones. The task consisted of listening in silence to a word list containing words with initial /p/. The participants were then asked to read target items visually presented on a computer screen. Nielsen, similar to Shockley et al., found that participants' VOTs for the consonants /p/ and /k/ were longer than baseline after listening to the word list with artificially lengthened VOTs. However, the same pattern did not occur after listening to the shortened VOTs. In that condition, participants' post-listening productions did not differ from baseline.

While these studies demonstrate that participants are able to modify their VOTs in an imitation task, they also suggest that there are specific limitations in the flexibility of the imitations. These limitations are especially crucial in the context of conversational interactions between interlocutors with differing linguistic backgrounds. For instance, languages sometimes differ in the length of VOTs of voiced and voiceless stops. Conversational convergence between interlocutors with such VOT differences in their native languages would require an imitation of artificially long or short VOTs with respect to their native phonetic categories (similar to Nielsen, 2011). Therefore, this paradigm is useful in approximating the task of a non-native speaker in a conversational interaction with a native speaker.

In the present study, we examine Spanish and English speakers' ability to imitate consonant-vowel tokens that vary in VOT, corresponding to their native and non-native phonemic categories, and in the length of the vowel following the consonant. For that, we used manipulated versions of the syllables /pa/ and /ba/. In both Spanish and English, unvoiced stop consonants (/p/, /t/, /k/) differ from their voiced counterparts (/b/, /d/, /g/) in VOT (Lisker and Abramson, 1964). However, in syllable initial position, the VOT values for Spanish [b] are negative (pre-voicing) while Spanish [p] is characterized by a short positive VOT. In English, [b] is characterized by short positive VOT (similar to the Spanish [p]), whereas [p] is characterized by a long lag VOT and aspiration. To compare Spanish and English speakers' ability to imitate both native and non-native tokens our stimuli are drawn from a prevoiced to long lag VOT continuum that encompasses both the native and non-native regions of each group. Participants were instructed to imitate the tokens to the best of their ability and we measured the VOTs and vowel lengths produced by the participants to assess how accurately they reproduced the characteristics of the token. While the specific ability of each group to accurately imitate the VOTs of the continuum members is unclear, both Nielsen (2011) and Shockley et al.'s (2004) work suggest that speakers are able to vary the voicing characteristics of syllable initial consonants in some circumstances. In addition to varying VOTs, the tokens also vary in the length of the vowel following the initial consonant. Participants' abilities to imitate varying vowel lengths will indicate whether they attend to duration information in the presented tokens. If, for example, there are no changes in VOT imitation across multiple tokens, but produced vowel length varies, this would imply that participants were attending to the token and attempting to imitate, but that they were either unable to distinguish VOT differences or were unable to produce them.

Furthermore, differences between Spanish and English listeners on their vowel length imitation may be interesting because of documented phonological regularities in English. Specifically, English is marked by a systematic variation in the length of vowels following voiced and voiceless consonants: vowels after voiced consonants tend to be longer than those after voiceless consonants (Allen and Miller, 1999). If English speakers are less able to imitate the VOT or vowel length of tokens that violate this regularity (i.e., short vowels following voiced consonants), then it would imply that the phonological regularities of their language are shaping their ability to imitate the tokens. This relationship is not strong or consistent in Spanish (see, Zimmerman and Sapon, 1957).

In summary, we predict that Spanish speakers and English speakers will differ on their ability to imitate different members of the VOT continuum. Specifically, Spanish speakers may have more difficulty than English speakers in producing tokens that are very unlike Spanish tokens and vice versa. Additionally, we expect that the accuracy of English speakers' imitations of either VOT or VL will be affected by whether the presented token violates the phonological regularities of English. We expect that Spanish speakers' imitation accuracy will not be affected by the VOT-VL relationship of the token. Finally, we predict that Spanish speakers and English speakers will be equally accurate in imitating token vowel length.

Examining Spanish and English speakers' performances on the imitation task will provide information on whether and how they differ in their ability to imitate native and non-native tokens. It may also provide information about the probability of obtaining social phonetic convergence in short term interactions between members of these two groups. That is, if, for example, imitation of non-native tokens proves extremely difficult for participants, it may indicate that any convergence seen between members of the two groups may not occur on the tokens we test. Conversely, if participants seem to be quite flexible on their imitations of these tokens, examination of stop consonants for researchers interested in social phonetic convergence may provide a good indication of whether convergence occurred.

## Methods

### Participants

Seventeen native speakers of Spanish (10 females, 7 males) who were students at Universidad Autónoma de Madrid and fifteen native English speakers (8 females, 5 males, 2 unreported) who were students at State University of New York - New Paltz participated in our study. Participants at SUNY - New Paltz received course credit for their participation. Participants at Universidad Autónoma de Madrid received a €5 discount ticket that could be used at the campus bookstore.

### Stimuli

A 20-year-old female native speaker of American English recorded multiple tokens of the diphone /ba/. The recordings were made in a quiet room using a Shure S58 stand microphone placed on the desk in front of the speaker. Recordings were made using Praat speech analysis software. The sampling rate was 44.1 kHz. A single token of [ba] (0 ms VOT, 275 ms vowel length) was then used to make an 11 step VOT continuum ranging from −60 ms VOT to +60 ms VOT. The 11 members of the continuum were each followed by three vowel lengths (175, 225, and 275 ms) for a total of 33 tokens. The procedure for creating the tokens is detailed below.

#### Consonants

In order to create the long lag VOT tokens, a 60 ms sample of aspiration from the same speaker (taken from a natural [p^h^a]) was inserted between the stop burst and the onset of voicing. Steps were created by removing 10 ms segments from the middle of the aspiration. Prevoiced consonants were created by taking a small sample of natural prevoicing from the same speaker (spontaneously present in one utterance of [ba]). The sample was copied and concatenated to create 60 ms of prevoicing. From the 60 ms of prevoicing, 10 ms segments were removed from the middle to create the continuum. To create the 5 ms prevoiced and VOT tokens, 5 ms of the prevoicing and VOT from the 10 ms step were removed respectively. This resulted in the following VOT conditions, ranging from −60 ms (prevoicing) to +60 ms (long lag VOT): ±60, ±50, ±40, ±30, ±20, ±10, ±5, 0. However, tokens of ±50 and ±40 were excluded to create an 11-step VOT continuum. This was done to shorten the duration of the overall experiment while still maintaining a concentration of VOT tokens around the middle of the continuum (−20 ms to + 20 ms), the region where both groups have a category boundary. The uneven sampling of the continuum is consistent with previous perceptual studies (Mann and Repp, 1981; Viswanathan et al., 2010) wherein it did not affect the perceptual performance of the listeners.

#### Vowels

The tokens were followed by three lengths of the vowel /a/.

We used the natural token of 275 ms as the long vowel in our stimuli. To create the short vowels, a 100 ms segment of the vowel was excised from the steady state portion of each CV continuum step. Similarly, to create the medium length vowels, a 50 ms segment was removed from the steady-state portion of each CV continuum member. The centers of the excised portions in the short and medium vowels were aligned. This resulted in a total of 3 different vowel length conditions: 175, 225, and 275 ms.

From these two manipulations we obtained 33 different stimuli (a combination of three vowel length and 11 VOT conditions). In a pilot study, two native Spanish speakers and two native English speakers were asked to freely categorize each token. All four pilot participants indicated that each continuum member was either a /pa/ or a /ba/. After the pilot task, participants were asked about the quality of the recordings and the ease of categorization. None of the participants reported difficulty with the tokens. In addition, over 99% of subjects' productions during the imitation of the continuum members in the main experiment were either a /pa/ or /ba/, further confirming the adequacy of the continuum.

### Procedure

Participants began the experiment by answering a series of questions about their linguistic background. Following this, they performed the imitation task. In each trial, participants listened to one of the 33 (11 VOT × 3 vowel length) tokens and were instructed to imitate the token they heard to the best of their ability. The speech syllables were presented through Sennheiser 555 headphones run through a Behringer HA400 micro amplifier at 70 dB SPL. Participants' imitations of the tokens were captured by a Shure S58 stand microphone placed on the desk in front of them in a quiet room. Recordings were made at a sampling rate of 44.1 kHz using the audio-editing software Audacity. Audio recordings of the entire session were saved as .wav files for later measurement. Presentation of the tokens occurred in four blocks with each block containing the 33 tokens presented in random order. After completing each block, the participants were allowed a break prior to beginning the next block. Once the final block was completed, the researcher debriefed the participant. In total, the participants produced 132 tokens (33 imitated tokens × 4 blocks). Procedures at the two running locations were identical except that all instructions, forms, and debriefings were provided in English in the US and Spanish in Spain. It took participants less than 15 min to complete the experiment.

## Results

Three trained research assistants who were blind to condition measured the VOTs and vowel lengths of each token produced by the participants. Measurements were made using Praat speech analysis software and following a written protocol for measuring the tokens. VOT was measured from the beginning of the stop burst to the onset of voicing. If voicing began before the stop burst, the measurement was assigned a negative value. Vowel length was measured as the duration of the steady state of the vowel. In all cases, measurements were made by the measurers through visual examination of the amplitude waveform and the spectrogram, as well as listening to the token and selections to ensure accurate duration measurement. The productions of 32 participants (15 English speakers, 17 Spanish speakers) were divided among the three measurers. Measurer 1 (M1) measured the tokens of 11 Spanish speakers and 11 English speakers, M2 measured the tokens of 5 Spanish speakers and 5 English speakers, and M3 measured tokens from 3 Spanish speakers and 2 English speakers. Each pair of measurers measured at least one common participant. From these common participants correlation coefficients were calculated to ensure good agreement among the measurers. All correlation coefficients exceeded 0.85 indicating good agreement. The final data set consisted of all measurements from M1, measurements of eight participants made by M2, and measurements of two participants made by M3. A total of 4224 tokens were recorded (132 tokens × 32 participants); of those, 60 tokens (1.4%) were excluded because of lack of clarity, anomalies in the signal, or difficulty in obtaining accurate measurements. Finally, both measured VOT and measured VL were submitted to an 11 (token VOT) × 3 (token vowel length) × 4 (block) × 2 (native language) ANOVA to determine if the block (i.e., 1st, 2nd, 3rd, or 4th utterance) had any effect. There were no main effects of block and no interactions of block with the other variables. Therefore, measured VOT and measured VL were averaged from the four utterances leaving 132 VOT measures and 132 VL measures per participant. These averages were used as the dependent variables in all analyses.

**Figure 1:**
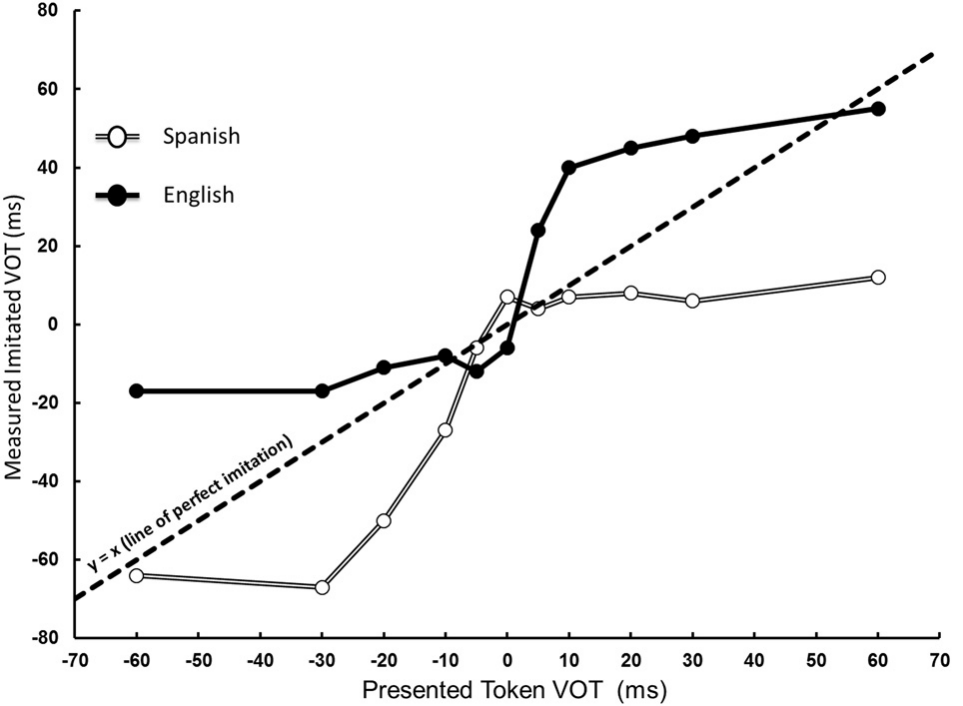
**Measured VOT as a function of token VOT and native language**. Spanish speakers (denoted by open circles) produced varying levels of prevoicing, within their native region, but did not demonstrate sensitivity in their non-native long lag region. Conversely, English speakers (denoted by filled circles) were unable to produce variations in their non-native prevoiced region, but produced varying levels of VOT in their native long lag region. The dotted line indicates perfect imitation performance.

Figure 1 shows measured VOT, henceforth referred to as produced VOT, as a function of token VOT for Spanish and English speakers. The *y* = x line in Figure 1 represents perfect imitation of the presented tokens. The measured VOT was submitted to an 11 (token VOT, within) × 3 (token vowel length, within) × 2 (native language, between) mixed ANOVA. The analysis revealed a main effect of token VOT, *F*_(10, 300)_ = 57.21, *p* < 0.001, η^2^_*P*_ = 0.656, indicating that, on average, participants varied their produced VOTs as a function of token VOT. A main effect of language, *F*_(1, 30)_ = 28.15, *p* < 0.001, η^2^_*P*_ = 0.484, indicated that the VOTs produced by Spanish speakers were different from those produced by English speakers. The analysis also showed an interaction between token VOT and native language, *F*_(10, 300)_ = 7.88, *p* < 0.001, η^2^_*P*_ = 0.21. This indicates that Spanish and English speakers differed in their productions of the different continuum members (Figure 1). Means and standard deviations of produced VOT for each level of token VOT for both Spanish and English speakers are presented in Table 1. There was no main effect of token vowel length (*F* < 1), vowel length did not interact with either token VOT or native language (*F* < 1, in both cases), and there was no three-way interaction, *F*_(20, 600)_ = 1.16, *p* = 0.28, η^2^_*P*_ = 0.037, indicating that token vowel length did not affect participant's produced VOTs. From Figure 1 it appears that the patterns of imitation of VOT, for the two language groups, are different for their respective native and non-native regions. To statistically evaluate this observation we performed polynomial trend analysis [see Holbert et al. (1990), for a review] for each group in the region of the continuum with negative VOT values (prevoicing) and, separately, in the positive VOT region of the continuum (voicing lag region). The imitations of the 0 VOT value were excluded from the analysis. In each analysis, the relationship between the token VOT and the produced VOT was examined. For the Spanish speakers, the trend analyses confirmed that while, in the native region, there was a linear (*p* < 0.001) and a quadratic relationship (*p* < 0.001), neither of these terms were significant in the non-native region [linear (*p* > 0.15) and quadratic (p > 0.5)]. None of the higher order terms were significant in either region for this group. Similar analyses were conducted for the English speakers in their respective native and non-native regions. Again, there was a systematic relationship between token and produced VOT in the native [linear (*p* < 0.001); quadratic (*p* < 0.05); cubic (*p* < 0.05)], but not in the non-native region [linear, quadratic, and cubic (*p* > 0.1)]. This pair of findings confirms the pattern in Figure 1 that both groups were substantially different in the imitation of their respective native and non-native regions of the continuum. The finding that both linear and non-linear terms were significant in the native region for both groups indicates that while subjects were sensitive to VOT changes in the presented tokens, perhaps their perceptual categories also influenced their imitation performance. This suggestion requires further empirical evaluation through a combination of perceptual and imitative tasks with the same stimuli.

**Table 1:** **Means and standard deviations (in ms) of produced VOT by token VOT for Spanish and English speakers**.

**Token VOT (ms)**	**Spanish**	**English**
−60	−64 (43)	−17 (29)
−30	−66 (35)	−17 (29)
−20	−49 (31)	−11 (31)
−10	−27 (30)	−8 (24)
−5	−6 (20)	−12 (34)
0	7 (12)	−6 (29)
5	4 (11)	24 (17)
10	7 (12)	40 (16)
20	7 (16)	45 (14)
30	6 (18)	48 (13)
60	11 (18)	55 (15)

**Figure 2:**
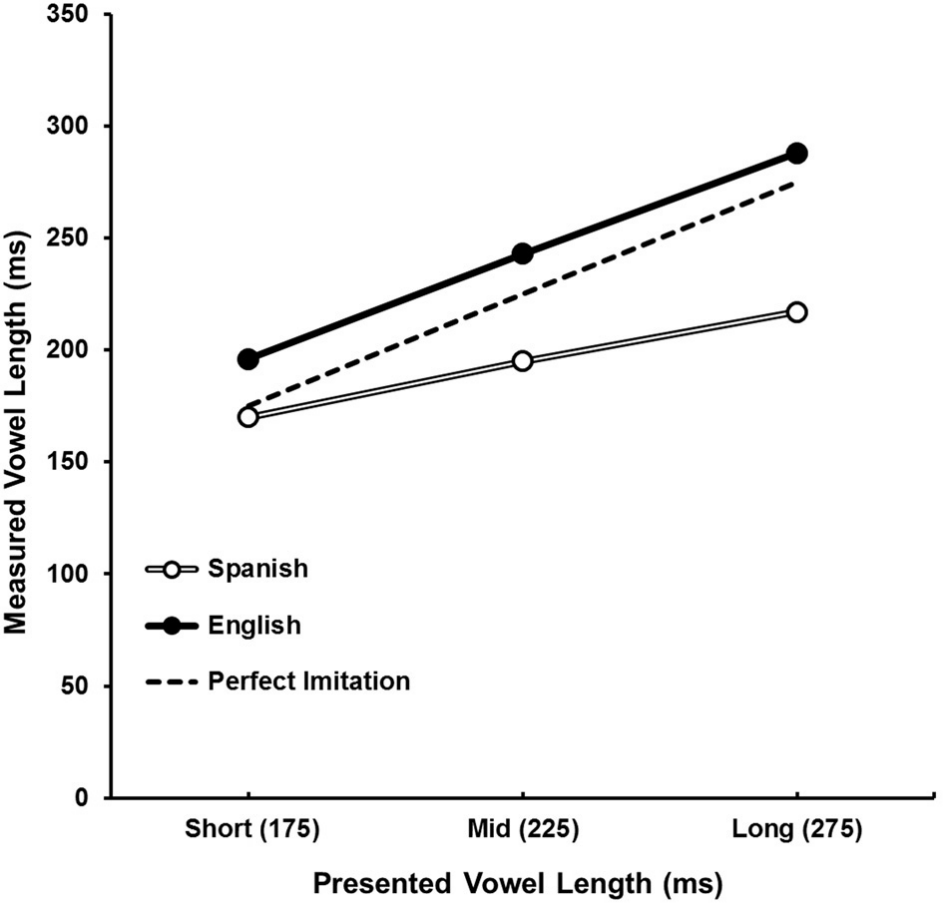
**Measured vowel length as a function of presented vowel length and native language**. Both Spanish (open circles) and English (filled circles) speakers vary their vowel length as a function of presented vowel length. However, English speakers consistently produce longer vowels than Spanish speakers. The dotted line indicates perfect imitation performance.

The vowel length imitation performance as a function of token vowel length for both Spanish and English speakers is depicted in Figure 2. The produced vowel length was submitted to an 11 (token VOT, within) × 3 (token vowel length, within) × 2 (native language, between) mixed ANOVA. Results indicate a main effect of token vowel length, *F*_(2, 60)_ = 38.62, *p* < 0.001, η^2^_*P*_ = 0.56, showing that produced vowel lengths changed as a function of token vowel length. A main effect of native language, *F*_(1, 30)_ = 10.08, *p* < 0.01, η^2^_*P*_ = 0.25, indicates that on average Spanish speakers' produced vowel lengths were different from English speakers'. Examination of the means indicates that Spanish speakers' vowels were shorter (*M* = 194 ms, *SD* = 77) than English speakers' (*M* = 243 ms, *SD* = 69). There was also a significant interaction between token vowel length and native language, *F*_(2, 60)_ = 4.02, *p* < 0.05, η^2^_*P*_ = 0.12. Examination of this interaction indicates that Spanish speakers produced smaller differences between the different levels of token vowel length (*M*1 = 170 ms, *SD* = 75; *M*2 = 195 ms, *SD* = 67; *M*3 = 217 ms, *SD* = 80) than did English speakers (*M*1 = 196 ms, *SD* = 51; *M*2 = 243 ms, *SD* = 60; *M*3 = 288 ms, *SD* = 61) (Figure 2). There was no main effect of token VOT, *F*_(10, 300)_ = 1.59, *p* = 0.11, η^2^_*P*_ = 0.05, and token VOT did not interact with either token vowel length, *F*_(20, 600)_ = 1.07, *p* = 0.38, η^2^_*P*_ = 0.03, or language, *F*_(10, 300)_ = 1.02, *p* = 0.429, η^2^_*P*_ = 0.03. This indicates that token VOT did not affect participants' productions of vowel length.

In summary, both groups' VOT imitations were substantially different in their native and non-native regions of the continuum. Additionally, both English and Spanish speakers altered produced vowel length depending on token vowel length. However, English speakers consistently produced longer vowels in all vowel length conditions compared to Spanish speakers. Finally, for neither group was there an influence of token vowel length on VOT imitation or of token VOT on vowel length imitation.

## Discussion

Native speakers of Spanish and English were compared on their abilities to imitate 11 members of a VOT continuum and three vowel lengths. Both groups of speakers produced VOTs that demonstrated sensitivity to within category differences in their native region of the VOT continuum (prevoiced region for Spanish and long lag region for English), but did not produce significant differences between tokens in their respective non-native regions. Spanish speakers systematically produced shorter vowels than English speakers for each level of vowel length. However, both groups varied their produced vowel lengths to follow the token vowel lengths. The VOT-vowel length regularity found in English did not seem to affect that group's ability to imitate the continuum tokens. This is indicated by the lack of effect of token vowel length on produced VOT and the lack of interaction with any other factor. The same pattern is seen for produced vowel length, i.e., there were no effects of token VOT on that variable.

While it is important to note that these results do not demonstrate phonetic convergence directly, they may help to understand findings that show phonetic convergence is diminished when interlocutors do not have similar language backgrounds. While the current investigation focuses on a different set of languages (Spanish and English instead of Korean or Chinese and English), the findings offer support to Kim et al.'s (2011) interpretation that convergence does not occur between linguistically distant interlocutors because their phonetic repertoires are different. Our participants have clearly different patterns of imitation showing that variations of native phonetic characteristics are both perceivable and producible^1^, (thus, are in the phonetic repertoire), but that variations in the non-native regions of the continuum are not in the repertoires of either group. Importantly, it is difficult to ascertain from our task whether the imitation performance in non-native regions stems from an inability to perceptually discriminate the non-native tokens or an inability to produce these tokens.

The current results differ from studies that have previously examined VOT imitation. For example, Nielsen (2011) found that English speakers' VOT productions did not differ from baseline when they heard tokens with shortened VOTs. In contrast, our English speakers appear to have successfully imitated tokens with shorter VOTs than those of typical voiceless tokens. Moreover, this ability was mirrored by the Spanish speakers who produced shorter durations of prevoicing than are typical suggesting that this ability applies broadly to voicing characteristics. While we did not collect baseline recordings from our participants, and, therefore, cannot definitively say that the tokens they produced are shorter than their typical productions, previous work has shown that American English VOTs for /p/ are generally around 60 ms and Spanish prevoicing for /b/ is around 120 ms (Lisker and Abramson, 1964). Thus, many of our tokens are shorter than the measured canonical tokens of /p/ and /b/ in the languages of our participants. Our results also differ from those reported by Flege and Eefting (1988). These authors examined imitations of a prevoiced /da/ to long-lag /ta/ continuum by Spanish and English monolingual adults and children, and bilingual adults and children. We focus on the results with monolingual adults because they are of direct relevance to our study. While the focus of their study differed considerably from the current work in that the continuum used represented a considerably larger range of VOTs allowing focus on between and within phoneme category differences, the imitation results can still be compared, although conservatively. In Flege and Eefting's work, Spanish speakers appear to exhibit prevoicing of a fairly constant duration even when imitating tokens that have 0 and 10 ms VOTs. This pattern is not evident in the current results. Additionally, English speakers' imitations of the short-lag region (0 to 30 ms) exhibit a constant VOT duration around 20 ms. Again, this pattern differs from the current findings. Interestingly, Flege and Eeftings work also shows that English speakers do not routinely imitate prevoicing. In the current study, English speakers on average produced prevoicing when imitating prevoiced tokens. The disparity may be explainable by the differences in methodology in the two studies. For example, in Flege and Eefting's work, participants heard a single token, categorized it, and then imitated the same token (without hearing it again). It is possible that this led to participants producing the category they had chosen instead of imitating the presented token. In contrast, in the current study, participants were not asked to categorize the tokens within the imitation task. Instead, the sole focus was on the imitation of what was heard.

Our results for vowel length may also support the importance of phonetic repertoire to phonetic convergence. While participants all followed the pattern of the token vowel length in imitating, consistent with past findings, English speakers' vowels were consistently longer than those of Spanish speakers (e.g., Fox et al., 1995). This pattern may have occurred because our token vowel lengths were chosen based on the productions of a model who is a native English speaker. Perhaps Spanish speakers' undershoot in vowel length was a result of having to imitate vowels that were simply longer than those they would normally produce. We advance this suggestion given that Spanish speakers in our study consistently produced shorter vowels than English speakers in all three vowel duration conditions. Again, this is difficult to confirm without baseline productions.

Finally, it has been suggested that interlocutors in social situations do not converge to items that are outside of their native phonetic space (Babel, 2009). This suggests that while speakers show flexibility with tokens of their native categories, this flexibility is limited within non-native categories. The current study provides clear evidence for this explanation with Spanish and English speakers. Furthermore, short-term phonetic convergence within social conversations is cited as a driver of long-term accent changes in non-native speakers of an ambient language (Pardo, 2006). The current study suggests that differences in interlocutors' phonetic repertoires place constraints on how they imitate phonetic information. These constraints likely extend to social interactions in which phonetic convergence may occur and to patterns in long-term accent change.

### Conflict of interest statement

The authors declare that the research was conducted in the absence of any commercial or financial relationships that could be construed as a potential conflict of interest.

## Footnotes

^1^The non-linear terms that are significant in the post-hoc analysis imply that there are likely constraints on imitation of tokens even within participant's native region of the continuum. This pattern may be attributable to phonemic category boundaries.
